# The Impacts of Prescribed Fire on PM_2.5_ Air Quality and Human Health: Application to Asthma-Related Emergency Room Visits in Georgia, USA

**DOI:** 10.3390/ijerph16132312

**Published:** 2019-06-29

**Authors:** Ran Huang, Yongtao Hu, Armistead G. Russell, James A. Mulholland, M. Talat Odman

**Affiliations:** School of Civil and Environmental Engineering, Georgia Institute of Technology, Atlanta, GA 30332, USA

**Keywords:** prescribed fire, burn activity, air quality modeling, CMAQ, decoupled direct method, respiratory effects

## Abstract

Short-term exposure to fire smoke, especially particulate matter with an aerodynamic diameter less than 2.5 µm (PM_2.5_), is associated with adverse health effects. In order to quantify the impact of prescribed burning on human health, a general health impact function was used with exposure fields of PM_2.5_ from prescribed burning in Georgia, USA, during the burn seasons of 2015 to 2018, generated using a data fusion method. A method was developed to identify the days and areas when and where the prescribed burning had a major impact on local air quality to explore the relationship between prescribed burning and acute health effects. The results showed strong spatial and temporal variations in prescribed burning impacts. April 2018 exhibited a larger estimated daily health impact with more burned areas compared to Aprils in previous years, likely due to an extended burn season resulting from the need to burn more areas in Georgia. There were an estimated 145 emergency room (ER) visits in Georgia for asthma due to prescribed burning impacts in 2015 during the burn season, and this number increased by about 18% in 2018. Although southwestern, central, and east-central Georgia had large fire impacts on air quality, the absolute number of estimated ER asthma visits resulting from burn impacts was small in these regions compared to metropolitan areas where the population density is higher. Metro-Atlanta had the largest estimated prescribed burn-related asthma ER visits in Georgia, with an average of about 66 during the reporting years.

## 1. Introduction

The World Health Organization’s International Agency for Research on Cancer classifies outdoor air pollution as carcinogenic to humans [1]. Particulate matter (PM) is a major component of air pollution that comes from both primary (e.g., dust, biomass burning, coal-fired power plant) and secondary (e.g., reactions of chemicals emitted from primary sources) sources [2]. Biomass burning is one of the most important sources of PM, but others include gases and aerosols, such as carbon monoxide, carbon dioxide, PM_2.5_ (PM with an aerodynamic diameter less than 2.5 µm) and black carbon. Biomass burning products, generally referred to as “smoke”, can reduce visibility and have adverse health effects. Epidemiological studies have shown associations between short-term PM_2.5_ exposure from fires and health endpoints, such as mortality, respiratory effects, and cardiovascular effects [3,4,5,6,7,8]. Wildfires do not only endanger lives and property, but also threaten public health with their large emissions of PM_2.5_ and other pollutants. Prescribed burning, another type of biomass burning, is a land management practice used to improve native vegetation and wildlife habitat, control insects and disease, and reduce wildfire risk [9]. In the U.S., prescribed burning is very popular, but its practice entails air pollution concerns. Biomass burning emissions remain as one of the largest sources of PM_2.5_ in the U.S., with an estimated 14% of total primary PM_2.5_ emissions coming from prescribed burning, while wildfires account for 16% [10]. Land managers who use prescribed burning to reduce damage due to wildfires must be mindful of the exposures resulting from prescribed burning. One advantage of using prescribed burning in this context is that burns can be planned to minimize adverse human health effects.

Rappold et al. [11] found that over 40% of Americans live in areas affected by wildland fires with a moderate or high contribution to ambient PM_2.5_ concentrations. In the southeastern U.S., prescribed burning contributes about 30% of PM_2.5_ emissions, while only 3% of PM_2.5_ emissions come from wildfires. Georgia actively uses prescribed burning for land management, with an annual statewide total burned area of over one million acres [12], one of the highest rates in the southeastern U.S. An estimated 33% of the total PM_2.5_ emissions come from prescribed burning in Georgia [10]. People living in Georgia and other parts of the southeastern U.S. are more likely to experience high and frequent smoke exposure in comparison to those living in other parts of the country due to increasing prescribed burning emissions. Recent epidemiological studies in Atlanta addressed the relationship between source-specific PM_2.5_ exposures and acute health effects, such as respiratory disease [13,14,15,16] and cardiovascular disease [15]. Sarnat et al. [15] found positive associations between cardiovascular disease-related emergency room (ER) visits and same-day PM_2.5_ concentrations attributed primarily to prescribed forest burning. Krall et al. [14] also found evidence of positive associations of respiratory disease-related ER visits with biomass burning PM_2.5_. A better understanding of the contributions of prescribed burning to human health is important, especially for the people who are directly affected by prescribed burning.

Although the air quality and health impacts of wildland fires have been studied in the past [4,8,11], the impacts specifically related with prescribed burning have not yet been evaluated. In this paper, we used prescribed burning permit information from the Georgia Forestry Commission and the BlueSky framework [17] to estimate the prescribed burning emissions of PM_2.5_ in Georgia during the first four months (January–April are the most active burning months in Georgia) of 2015–2018. We then used the Community Multiscale Air Quality (CMAQ) model [18], a chemical transport model, with the decoupled direct method (DDM) for source-specific impact estimation [19] to compute the contribution of prescribed burning on simulated PM_2.5_ concentrations. Based on the data fusion method of Friberg et al. [20], we merged these emission-based air quality model simulation results with observations at monitoring sites to provide spatiotemporal PM_2.5_ exposure fields for use in health impact estimations. Finally, we employed a general health impact function to estimate the impacts of prescribed burning on asthma-related ER visits in Georgia during the first four months of 2015–2018.

## 2. Materials and Methods

### 2.1. Burn Impact Exposure Fields

**Figure 1:**
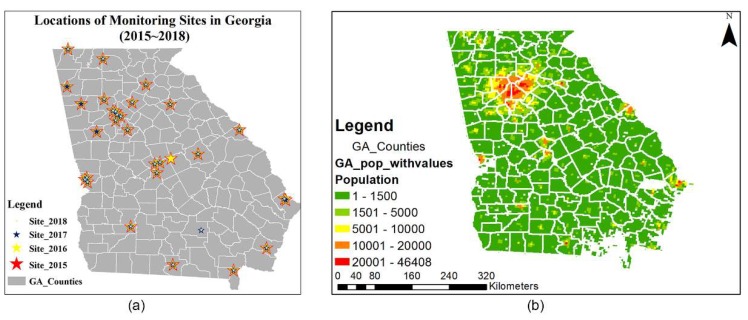
(**a**) Locations of monitoring sites in Georgia: 2015 (red stars), 2016 (yellow stars), 2017 (blue stars), and 2018 (light green stars); and (**b**) distribution (at 4 km resolution) of Georgia’s population (9,687,653) according to the 2010 U.S. Census.

Fire emissions estimates were developed using the BlueSky framework [17] with location, date and burned area information from the Georgia Forestry Commission’s (GFC) burn permit database. Permit records do not contain accurate start times and durations for most burns. In a previous study [12] that focused on permit records and a phone call survey with typical burners, we found most burns started around 10 a.m. Meanwhile, per ordinance, all fires should be finished before sunset, which is around 5 p.m. during the winter in Georgia. For simplicity, we assumed all fires started at 10 a.m. local time and lasted 6 h. Emissions data for other sources, such as mobile, agricultural, and biogenic emissions, were projected from the 2011 National Emission Inventory (NEI) to the relevant year using forecasted meteorology and temporal parameters such as day of the week. The meteorology was forecasted one day earlier based on the latest observations using the Weather Research and Forecasting model (WRF, version 3.6, National Center for Atmospheric Research, Boulder, Colorado). Daily total PM_2.5_ concentrations for the first four months of 2015–2018 were simulated using the CMAQ (v5.0.2, U.S. Environmental Protection Agency, Research Triangle Park, NC, USA) model in a domain at 4 km spatial resolution over Georgia and surrounding states. These simulated PM_2.5_ concentrations were then fused with daily total PM_2.5_ observations at ambient monitors (Figure 1a) using the approach developed by Friberg et al. [20,21]. The resulting PM_2.5_ fields included spatiotemporal information of the observations, as well as the spatiotemporal completeness provided by the air quality model, with the data fusion process decreasing model biases and errors. The DDM sensitivity analysis tool embedded into CMAQ [22] was used to quantify the air quality impacts associated with prescribed burning. The observation-fused daily total PM_2.5_ fields were multiplied by the ratio of the burn impact to the total PM_2.5_ from CMAQ–DDM for each day and each grid cell to generate an “observation-adjusted burn impact” on PM_2.5_ (Equation (1)).

(1) $$AdjustedB_{i,j}^{t}=\frac{B_{i,j}^{t}}{C_{i,j}^{t}}\times DFC_{i,j}^{t}\;$$

where *i* and *j* are the horizontal column and row indices for the vertical column, t is the day, *B* is the burn impact from CMAQ–DDM (in µg/m^3^), *C* is the total PM_2.5_ concentrations from CMAQ–DDM (in µg/m^3^), *DFC* is the total PM_2.5_ concentration after applying the data fusion method (in µg/m^3^), and *AdjustedB* is the observation-adjusted burn impact (in µg/m^3^).

### 2.2. Health Impact Function

**Figure 2:**
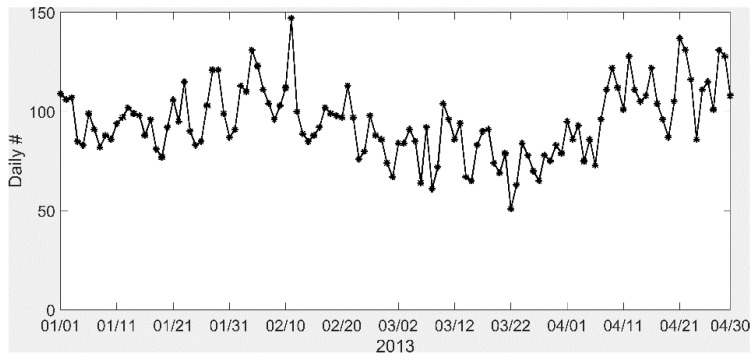
Observed daily emergency room (ER) visits for asthma in the Atlanta area (20 counties included) in 2013.

We used a log-linear relationship between air pollutant concentration change and health outcome incidence to quantify the health impact from prescribed burning as follows:

(2) $$\Delta Y=\;Y_{0}\left(1-e^{-\beta \Delta PM}\right)\times Pop$$

where $Y_{0}$ is the baseline incidence rate for the health endpoint, β is the health effect estimate from the epidemiological study, ΔPM is the change in air pollutant concentration, and Pop is the population exposed to the air pollution [23]. Here, we focused on the ER visits for asthma as the health endpoint due to a reported positive association between biomass burning PM_2.5_ and respiratory disease-related ER visits [14]. We used national asthma-related ER visits in 2013 (a rate of 625.6 per 100,000 people) as the annual asthma-related ER visit rate in Georgia for 2013 and converted to a daily rate by constructing weights based on the observed daily ER visits due to asthma during 2013 (Figure 2), as follows:

(3) $$weight\left(i\right)=\;\frac{ER\;visits,asthma\;count_{i}}{\sum_{i=1}^{365}ER\;visits,\;asthma\;count_{i}}$$

where $ER\;visits,asthma\;count_{i}$ is the number of ER visits due to asthma on day  i. Daily ER visits due to asthma in the Atlanta area for the first four months in 2013 had an average value of 94.8, with a standard deviation of 17.5. The asthma-related ER visit rate is about 669.8 per 100,000 people.

We used a health effect estimate of β=0.008 (0.004–0.012) for asthma-related ER visits that was derived from a wildfire smoke exposure epidemiological study [24]. ΔPM is the observation-adjusted burn impact on total PM_2.5_. We extracted the population from the Environmental Benefits Mapping and Analysis Program, Community Edition (BenMAP-CE) [25], and allocated the 2010 block-level U.S. Census population to the 4 km spatial resolution grid using the PopGrid program provided by the U.S. Environmental Protection Agency (EPA) (Figure 1b) [26]. While the levels of health impacts we present here are for the estimated asthma-related ER visits attributable to prescribed fire contribution to PM_2.5_, the spatial and temporal variations would be similar for other health impacts that can be formulated with Equation (2).

## 3. Results and Discussion

### 3.1. Total PM_2.5_ Concentrations and Fire Impact Exposure Fields from CMAQ and Data Fusion (DF)

**Figure 3:**
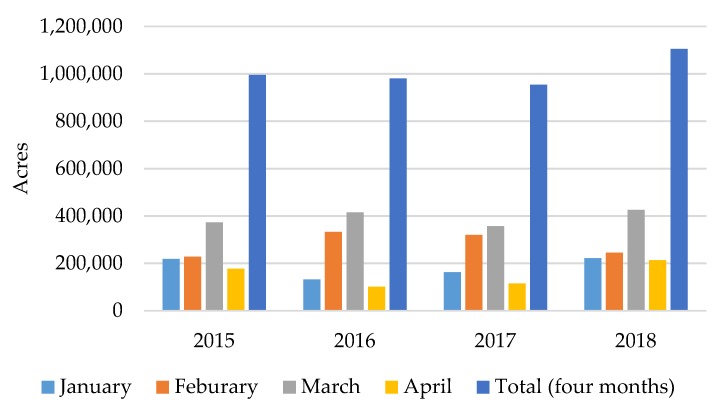
Total monthly burned area from 2015 to 2018 in the first four months (prescribed burning season; permit data from the Georgia Forestry Commission [GFC]).

Average PM_2.5_ concentrations over the four-year period from 2015 to 2018 during the prescribed burning season showed that the CMAQ simulations of total PM_2.5_ concentrations were biased low compared to the observations (Table 1). Fire impact increased slightly starting from 2016, although the 2017 burned area was the smallest among the four-year period (Figure 3). The drought in the southeastern U.S., which started in the fall of 2016 [27], may have led to larger fuel loads [28] and, consequently, to greater emissions per unit burned area. This, in turn, may have contributed to the larger fire impact in 2017. The spatial plots of the monthly averages for total PM_2.5_ and fire impact (Figures S1–S4) indicated that high concentrations in southwestern and east-central Georgia (GA) were mainly due to the active prescribed burning. January, February, and March were more active burn months than April. March was the most active burn month, accounting for about 40% of the total burned area among the first four months (Figure 3). The year of 2018 had a total burned area of over one million acres for the first four months, which resulted in the largest burn impact over the last four years (2015–2018).

Comparisons of daily total PM_2.5_ concentration between observations and the CMAQ model during January to April over four years had slopes less than 0.5 and R^2^ values in the range of 0.13 to 0.30 (Figure S5). Based on the recommended performance statistics used to assess photochemical model performance (24-h PM_2.5_ criterion: *R* (correlation coefficient) > 0.4) from Emery et al. [29], only the *R*^2^ of 2016 did not meet the criterion. All years’ normalized mean errors (NMEs) met the criterion (<50%) and goal (<35%) (Table 2). The criteria and goals in Emery et al. [29] are based on recent regional photochemical grid model applications in support of U.S. regulatory actions for PM_2.5_ and regional visibility, and are defined as follows: (1) Estimates meet the criteria if model performance is in the top 2/3 of all past applications; (2) estimates meet goals if performance is better than 2/3 of past model applications.

Comparisons of the CMAQ (Figure S5) and data fusion (DF) (Figure S6) estimates with observations (OBS) showed that the results improved after application of the data fusion method, with slopes ranging from 0.73 to 0.80 and *R*^2^ values between 0.74 and 0.85. Data fusion normalized root mean square errors (NRMSEs) decreased by 60% compared to CMAQ NRMSEs. NMEs also decreased and were close to zero (Table 2). We also completed a 10% data withholding evaluation. Ten groups of observational data were constructed, each group was run independently, with each group having 10% of the data randomly withheld. Performance was evaluated by comparing the simulated values to the data that were withheld for that group. The 10% data withholding (WH) results (Figure S7, Table 2) also had smaller NRMSEs and NMEs, and larger R^2^ compared to the CMAQ results. As expected, all those results showed better performance with the application of the data fusion method. Occasionally, simulated daily PM_2.5_ concentrations from CMAQ were larger than the observations. This was due to simulated fire impacts that were not captured by observations at the monitoring sites in the region, leading to large differences between the data-fused and original CMAQ results (Figures S1–S4). This could lead to the data fusion process decreasing the modeled impact of fires.

Application of our data fusion method to adjust fire impacts provides improved exposure fields for health analysis. However, limited observational data can lead to reduced fire impact estimates when fused with simulations, even though the smoke plume is captured by the simulations. A lack of monitoring sites can be alleviated, in part, by using low-cost sensors. However, performance of low-cost sensors is still unclear when applying them to detect impact from prescribed fires. Several studies show that some types of low-cost sensors have better performance in high concentration environments [30,31,32], but are less sensitive to changes in PM_2.5_ concentrations compared to a referenced instrument [33].

**Figure 4:**
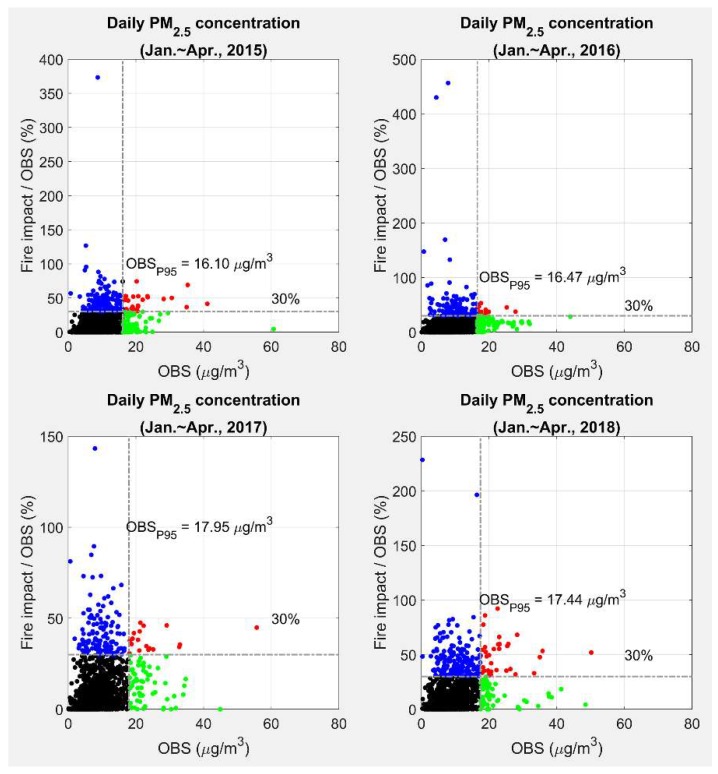
Relationship between daily total PM_2.5_ observations (OBS) and ratios of observation-adjusted fire impact to OBS during January–April from 2015 to 2018. The number of points are listed in Table 3.

We also analyzed the ratio of observation-adjusted burn impact to observed PM_2.5_ as a function of observed daily total PM_2.5_ (Figure 4 and Table 3). In Figure 4, the grey dashed lines represent the 95th percentile of observations (vertical) and 30% fire impact to observed PM_2.5_ (horizontal). The reason we chose 30% here was because about 30% of PM_2.5_ emissions came from prescribed burning in Georgia according to the 2014 NEI. Hence, the days with a ratio larger than 30% received a larger than average prescribed burning impact and may be seen as high fire impact days. The red dots in Figure 4 represent the days with high observed PM_2.5_ concentrations due to fire impacts. Future epidemiological studies could focus on such days to find a relationship between short-term high-level PM_2.5_ exposures due to fire and health impacts over a series of single-day lags. On the other hand, the blue dots are the days when the prescribed fire was still a major source of total PM_2.5_. However, due to the low observed PM_2.5_ concentrations, while concerns cannot be raised about air quality on those days, health impacts from smoke may be non-negligible. Finally, the green dots are the high PM_2.5_ days, when other sources are more to blame than prescribed fire smoke. Nearly 13% of PM_2.5_ observations in 2018 had burn impacts larger than 30%, which was somewhat larger than previous years (Table 3).

### 3.2. Health Impacts from Prescribed Burning

**Figure 5:**
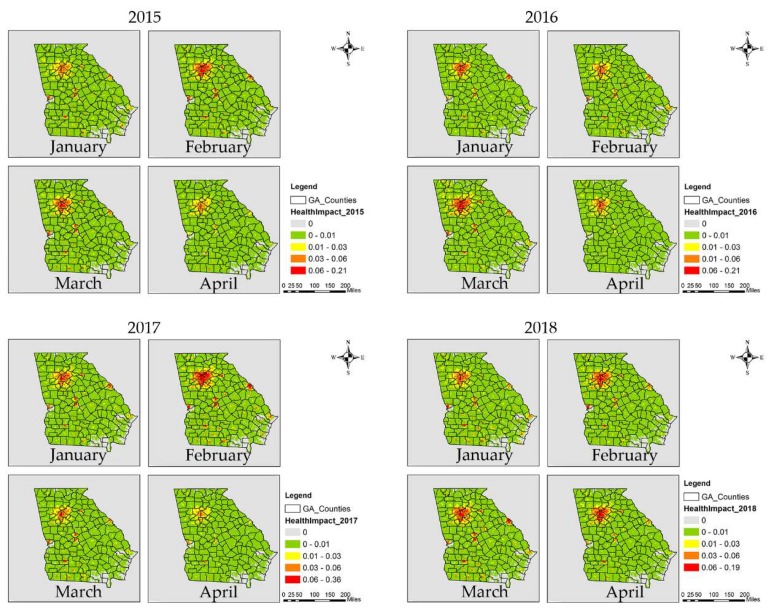
Estimated total monthly asthma-related ER visits (health impact) due to prescribed fires from 2015 to 2018, first four months.

Health impacts from prescribed fires during the first four months of 2015–2018, as reflected in the estimated monthly average asthma-related ER visits presumed to have resulted from the burns, showed spatial and temporal variation (Figure 5). Southwestern, central and east-central GA had large health impacts due to the intense prescribed burning activity. Macon metropolitan statistical area (MSA) (Bibb County), Atlanta MSA (Fulton, Gwinnett, DeKalb and Cobb Counties), Albany MSA (Dougherty County), Augusta MSA (Columbia and Richmond Counties), Warner Robins MSA (Houston County), Valdosta MSA (Lowndes County), and Columbus MSA (Muscogee County) had larger estimated health impacts in terms of absolute numbers due to both large populations and high levels of fire impact on PM_2.5_. Although prescribed burning had a relatively small impact on Atlanta MSA’s PM_2.5_ air quality (Figures S1–S4), the large population still led to a large estimated health impact. January, February, and March experienced the larger estimated health impacts due to more active prescribed burning (Figure 3). January 2015, February 2016 and March 2017 had lower estimated health impacts compared to the same months in other years, while February 2017 and March 2016 had higher estimated health impacts. The month with the largest and smallest health impact varied by year depending on meteorology and subsequent burn activity. Typically, April was not as active a burn month as others; however, April 2018 had more burns than February in central and east-central Georgia (Figures S1–S4), and those burns affected Atlanta MSA’s air quality. Also, April 2015 had more burns in southern Georgia, though larger fire impacts were limited to less populated regions.

**Figure 6:**
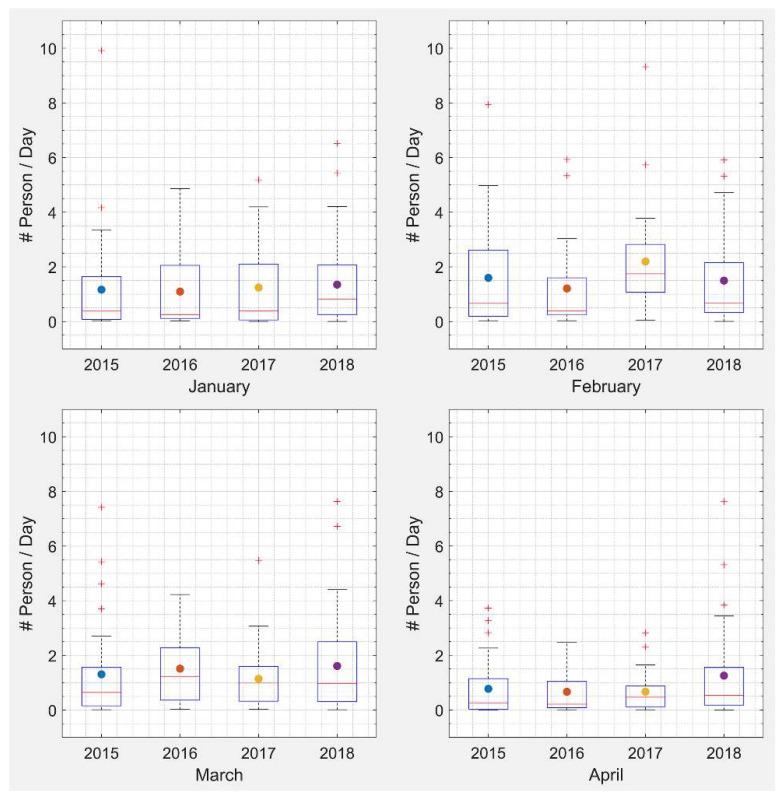
Estimated daily asthma-related ER visits in Georgia due to prescribed burning for January–April of each year from 2015 to 2018. The central mark indicates the median, the point indicates the mean, and the bottom and top edges of the box indicate the 25th and 75th percentiles, respectively. The whiskers bound the range of values excluding the outliers.

**Figure 7:**
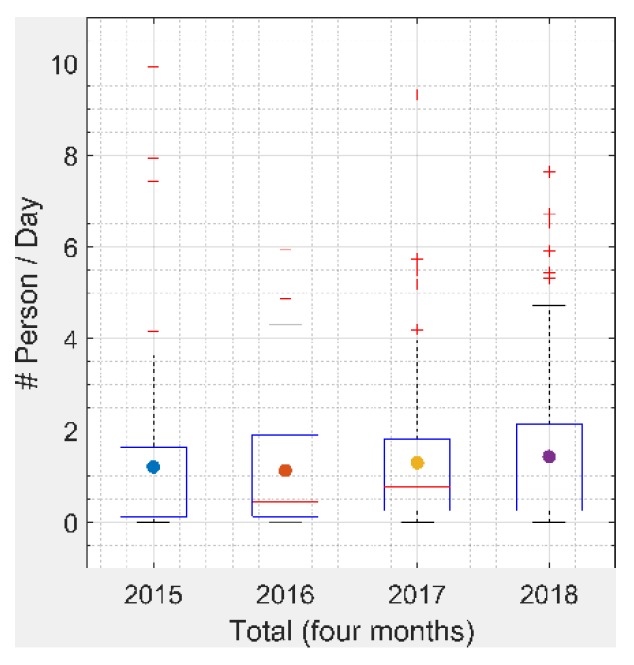
Estimated daily asthma-related ER visits in Georgia due to prescribed fires for the first four months of 2015–2018. The central mark indicates the median, the point indicates the mean, and the bottom and top edges of the box indicate the 25th and 75th percentiles, respectively. The whiskers bound the range of values, excluding the outliers.

Estimated daily asthma-related ER visits due to prescribed burning for each year showed that February and March had larger health impacts than January, with higher estimated daily health impacts (Figure 6). February 2017 had a larger estimated daily health impact compared to the other years, probably due to larger emissions from the drought, as discussed above. Since fire locations and metrological conditions were different each day, there may have been more burns affecting large populated areas in this month. April 2018 also had a larger estimated daily health impact with more burned areas compared to previous years, likely due to an extended burn season resulting from the need to burn more areas. There was a slightly increasing trend of estimated daily average health impact from 2016 to 2018 (Figure 7).

The estimated total health impacts increased from 2016 to 2018 (Table 4) as the prescribed burning season appeared to get longer. From 2015 to 2017, April had less health impacts compared to the first three months, but in 2018 the estimated total health impact of April doubled compared to previous years. Interestingly, although the burned areas in February 2015 and 2018 were about 60% of that of the corresponding March (Figure 3), the estimated total health impact of those two months in each of the two years was similar (Table 4). This result indicates that there were more populated areas (Atlanta MSA and Augusta MSA) affected by prescribed burning during February than March in 2015 and 2018. February 2017 had the largest estimated health impacts across the reporting years, with 62 ER visits due to asthma—a rate of 6.4 per 1,000,000 people. There was less difference in estimated total health impact among different months in 2018 compared to previous years. In the first four months of 2015, there were about 145 ER visits estimated to be due to asthma because of prescribed burning impact, a rate of 15 per 1,000,000 people. This increased by about 18% in 2018, compared to 2015. The estimated total number of ER visits increased by about 15% from 2016 to 2017, especially for February in 2017, with an increase of about 77%. Approximately 38 ER visits were associated with exposure to prescribed burning in April 2018, exhibiting an increase of over 60% compared to 2015 (23) and 90% compared to 2016 (20) and 2017 (20).

**Figure 8:**
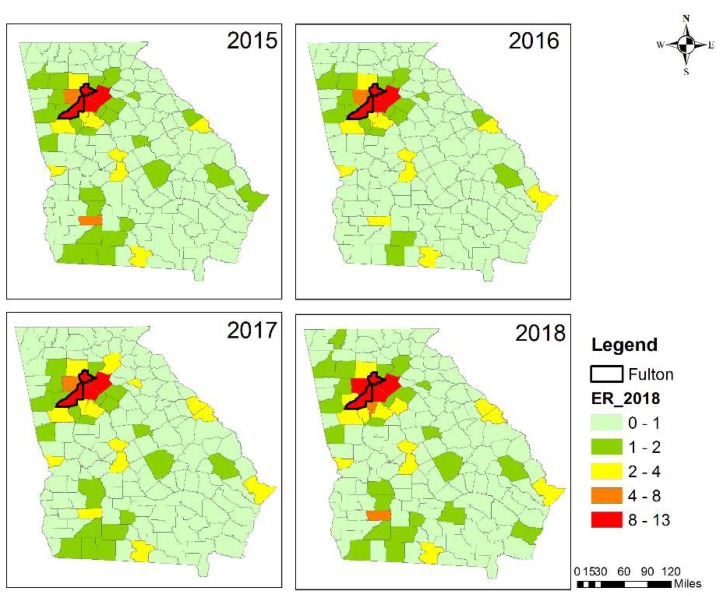
Estimated asthma-related ER visits by the county in Georgia from 2015 to 2018 for the first four months of the year.

**Figure 9:**
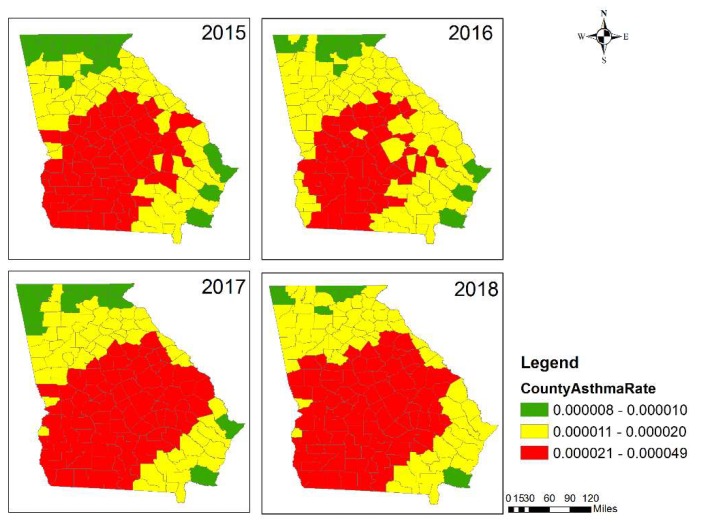
Estimated asthma-related ER visit rates (per person) due to prescribed burning by county in Georgia from 2015 to 2018 for the first four months of the year.

Fulton County had the largest estimated health impact due to the largest population (Figure 8). There were 10 ER visits estimated due to asthma because of prescribed burning impact in 2015 during the burn season. The number increased by about 30% to 13 in 2018. Gwinnett, DeKalb, and Cobb Counties, all within the Atlanta MSA, were the other three counties that had over 5 estimated ER visits due to an asthma issues during the burn season every year from 2015 to 2018 (Figure 8). The estimated asthma-related ER visits due to prescribed burning in the Atlanta MSA had an average of about 66 during the reporting years, which was about 0.58% of the total observed asthma-related ER visits (11,372) in the Atlanta MSA for 2013. Dougherty County had the largest estimated health impact rate in the southwestern GA (Figure 9), but only with an estimated average of about 4 people visiting the ER in relation to asthma during the burn season for the four years (2015–2018) due to the small population size. The Atlanta MSA had the highest number of people visiting the ER due to asthma caused by burn impact.

The health impact estimates here are subject to various uncertainties that should be considered. For example, the β parameter of the health impact function we used was derived from wildfire impact epidemiological studies. A lack of epidemiological studies to provide prescribed burning-specific concentration-response functions (β′s) is a weakness of the health analysis here. The β parameter may be different for prescribed fire specific cases. An epidemiological study that focuses on the Atlanta area [14] shows a positive association (relative risk = 1.006 (1.003–1.01)) between respiratory disease and biomass burning source. Considering the sources of PM_2.5_ in Georgia, the biomass burning is mainly prescribed burning emissions. Besides the uncertainty in β, there are other uncertainties in estimating the prescribed burning related health impact. Previous studies show that the measured PM_2.5_ emissions from prescribed burning is about 90% of modeled emissions [34]. Using the BlueSky framework (as done here), the PM_2.5_ emissions we estimated for the same case were about 60% of measured emissions. The horizontal and vertical allocation of fire emissions in the model could lead to about 20% uncertainty in the final pollutant concentration estimation [35]. Inaccurate simulated wind speed and wind direction may also bring uncertainty as high as 100% [36]. Fire duration is also difficult to determine due to a lack of post-fire information. Based on the NRMSE of the 10% data withholding (Table 2), the uncertainty of fire impact was about 40% among the reporting years. Changing the health impact function parameters (β,  ΔPM) would only change the level of health impact of prescribed burning and the exact number of impacted people, while the spatial and temporal variations of prescribed burning impact on public health would not change.

## 4. Conclusions

Data fusion of observed PM_2.5_ concentrations greatly improved the spatial and temporal accuracy of the PM_2.5_ exposure fields generated in simulations with CMAQ data. This was evidenced in the statistics of linear regressions between the data fusion results and observations: (1) Slopes that were less than 0.5 before ranged from 0.73 to 0.80 after data fusion, while (2) R^2^ values previously between 0.13 and 0.30 later ranged from 0.74 to 0.85. Even with 10% data withholding, errors were significantly reduced: (1) The normalized root mean square error decreased from approximately 60% to 40%, while (2) the normalized mean error dropped from approximately 25% to less than 5%.

In this paper, we have described a new method for deriving observation-adjusted prescribed fire impact from data-fused PM_2.5_ exposure fields. The method consists of scaling the data-fused PM_2.5_ fields with the ratio of DDM-calculated fire impact to CMAQ-computed PM_2.5_ concentration. Using observation-adjusted fire impact could help to identify days and areas with major prescribed fire impact on local air quality, even if the observations are low, and also distinguish those days from the other days when the fire impact is low. Such days and areas should be investigated further in epidemiological studies to find the relationship between health effects and prescribed burning.

Health impacts were formulated with a log-linear relationship between the health outcome and the contribution of prescribed fire to PM_2.5_ concentrations, and were represented by the asthma-related ER visits. Variations in prescribed burning activity during the first four months of 2015–2018 in Georgia led to temporal and spatial differences of health impacts. This points to the importance of distinguishing seasons and areas when studying prescribed fire and its health impacts. April 2018 had approximately 65% more burned areas and an 80% larger estimated total health impact than Aprils in previous years. This was likely due to an extension of the burn season in response to an increased demand for burning throughout Georgia.

Southern Georgia had the greatest prescribed burning activity and consequently the highest PM_2.5_ levels in the state. However, the largest health impacts, in terms of absolute number of asthma-related ER visits, with an average of about 66 people in the burn months during the reporting years, were found in the Atlanta MSA due to the much larger population exposed to moderate levels of prescribed burning emissions. In the less populated county of Dougherty in southeastern Georgia, the number of asthma-related ER visits was limited to 4, although the prescribed fire impact on PM_2.5_ levels was highest there.

## Figures and Tables

**Table 1:** Means and standard deviations (µg/m^3^) of the total particulate matter with an aerodynamic diameter less than 2.5 µm (PM_2.5_) concentrations and burn impacts at monitoring sites during the prescribed burning seasons (January–April) of 2015–2018.

PM_2.5_	2015	2016	2017	2018
Observation	8.6 ± 4.3	8.3 ± 4.4	8.8 ± 5.3	8.7 ± 5
CMAQ *	6.6 ± 4.5	6.1 ± 4.3	6.3 ± 4	6.2 ± 4
Burn impact (CMAQ)	0.83 ± 2.82	0.76 ± 2.96	0.87 ± 1.62	0.97 ± 2.26
Data fusion	8.4 ± 3.8	8 ± 3.7	8.7 ± 4.6	8.2 ± 4.2
Burn impact (data fusion)	0.91 ± 1.97	0.86 ± 1.77	1.06 ± 1.77	1.24 ± 2.42

* CMAQ: Community Multiscale Air Quality model.

**Table 2:** Normalized root mean square error (NRMSE) and normalized mean error (NME) of Community Multiscale Air Quality (CMAQ) model simulated and data-fused daily total PM_2.5_ concentrations with respect to observations during the first four months of each year (2015–2018).

PM_2.5_	NRMSE				NME			
	2015	2016	2017	2018	2015	2016	2017	2018
**CMAQ**	0.57	0.64	0.59	0.62	0.23	0.26	0.28	0.28
**DF**	0.23	0.27	0.24	0.24	0.02	0.04	0.01	0.05
DF (withholding) *	0.40	0.40	0.42	0.36	0.01	0.04	0.01	0.05

* DF (withholding): 10% data withholding

**Table 3:** Number of points for each quadrant in Figure 4.

Days	2015	2016	2017	2018
Total	2069	2136	1565	1762
High fire impact/High PM_2.5_ (Red)	27	14	18	36
High fire impact/Low PM_2.5_ (Blue)	141	124	133	192
Low fire impact/High PM_2.5_ (Green)	75	93	60	52
Low fire impact/Low PM_2.5_ (Black)	1826	1905	1354	1482

**Table 4:** Estimated total monthly ER visits due to asthma in Georgia. The uncertainties were derived from Equation 2 assuming 50% uncertainty in β (the health effect estimate from the epidemiological study) and 40% uncertainty in ΔPM (the change in air pollutant concentration).

Year	January	February	March	April	Total
**2015**	36 ± 23	45 ± 27	41 ± 25	23 ± 16	145 ± 46
**2016**	34 ± 21	35 ± 20	47 ± 22	20 ± 14	136 ± 39
**2017**	39 ± 21	62 ± 30	35 ± 19	20 ± 11	156 ± 43
**2018**	42 ± 24	42 ± 23	50 ± 29	38 ± 26	171 ± 51

## Supplementary Materials

The following are available online at https://www.mdpi.com/1660-4601/16/13/2312/s1, Figure S1: January–April monthly averages of total PM_2.5_ and fire impact (2015): CMAQ-simulated, data fused and their difference; Figure S2: January–April monthly averages of total PM_2.5_ and fire impact (2016): CMAQ-simulated, data fused and their difference; Figure S3: January–April monthly averages of total PM_2.5_ and fire impact (2017): CMAQ-simulated, data fused and their difference; Figure S4: January–April monthly averages of total PM_2.5_ and fire impact (2018): CMAQ-simulated, data fused and their difference; Figure S5: Comparison of daily total PM_2.5_ concentration between observations (OBS) and CMAQ from 2015 to 2018, first four months; Figure S6: Comparison of daily total PM_2.5_ concentration between observations (OBS) and DF from 2015 to 2018, first four months; Figure S7: Comparison of daily total PM_2.5_ concentration between observations (OBS) and 10% data withholding DF from 2015 to 2018, first four months.
