# Supplementary material for: The Impacts of Prescribed Fire on PM2.5 Air Quality and Human Health: Application to Asthma-Related Emergency Room Visits in Georgia, USA
Source: Int J Environ Res Public Health. 2019 Jun 29;16(13):2312. doi: 10.3390/ijerph16132312 (PMC6651061; doi:10.3390/ijerph16132312)
Supplement: Supplementary file 1 [file ijerph-16-02312-s001.pdf]

*International Journal of Environmental Research and Public Health*

Supplemental Material for

# The Impacts of Prescribed Burning on $PM_{2.5}$ Air Quality and Human Health: Application to Georgia, USA

**Ran Huang, Yongtao Hu, Armistead G. Russell, James A. Mulholland, M. Talat Odman ***

School of Civil and Environmental Engineering, Georgia Institute of Technology, Atlanta, GA 30332, USA; ranhuang2014@gatech.edu (R.H.); yh29@mail.gatech.edu (Y.H.); ted.russell@gatech.edu (A.G.R.); james.mulholland@ce.gatech.edu (J.A.M.)

* Correspondence: odman@gatech.edu

**Contents of this file**

Figures S1 to S7

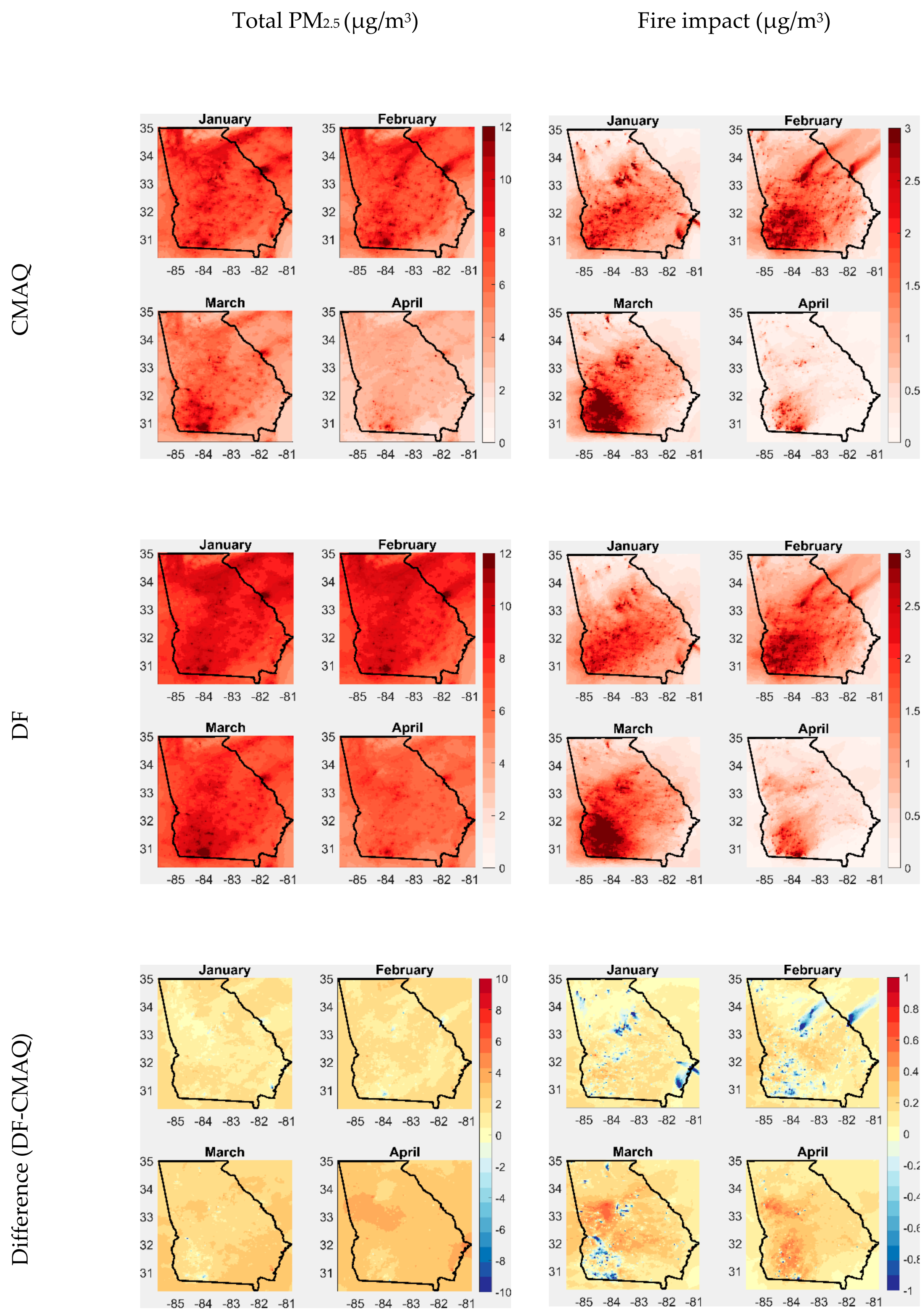


**Figure S1.** January–April monthly averages of total $PM_{2.5}$ and fire impact (2015): CMAQ-simulated, data fused and their difference.

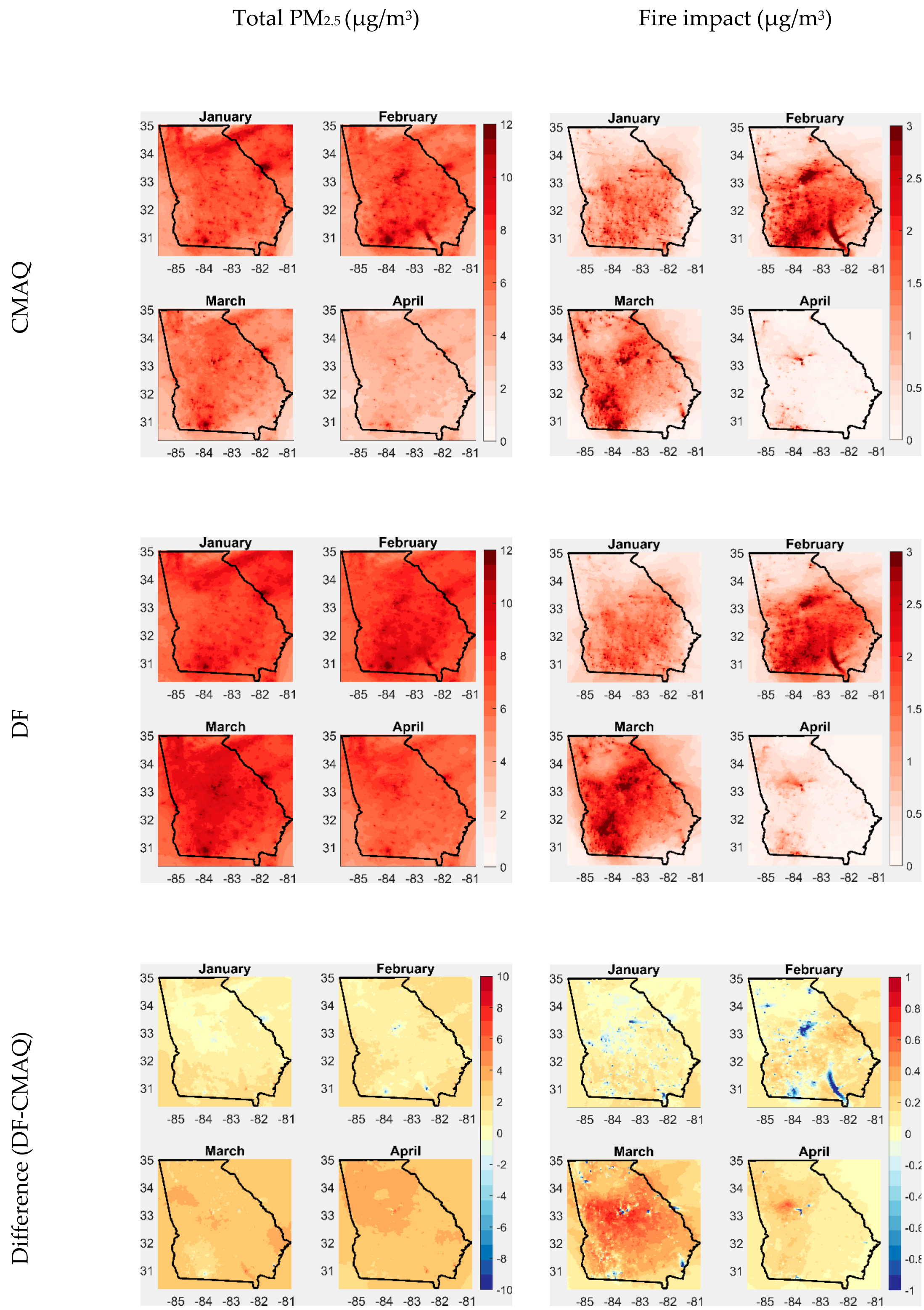


**Figure S2.** January–April monthly averages of total $PM_{2.5}$ and fire impact (2016): CMAQ-simulated, data fused and their difference.

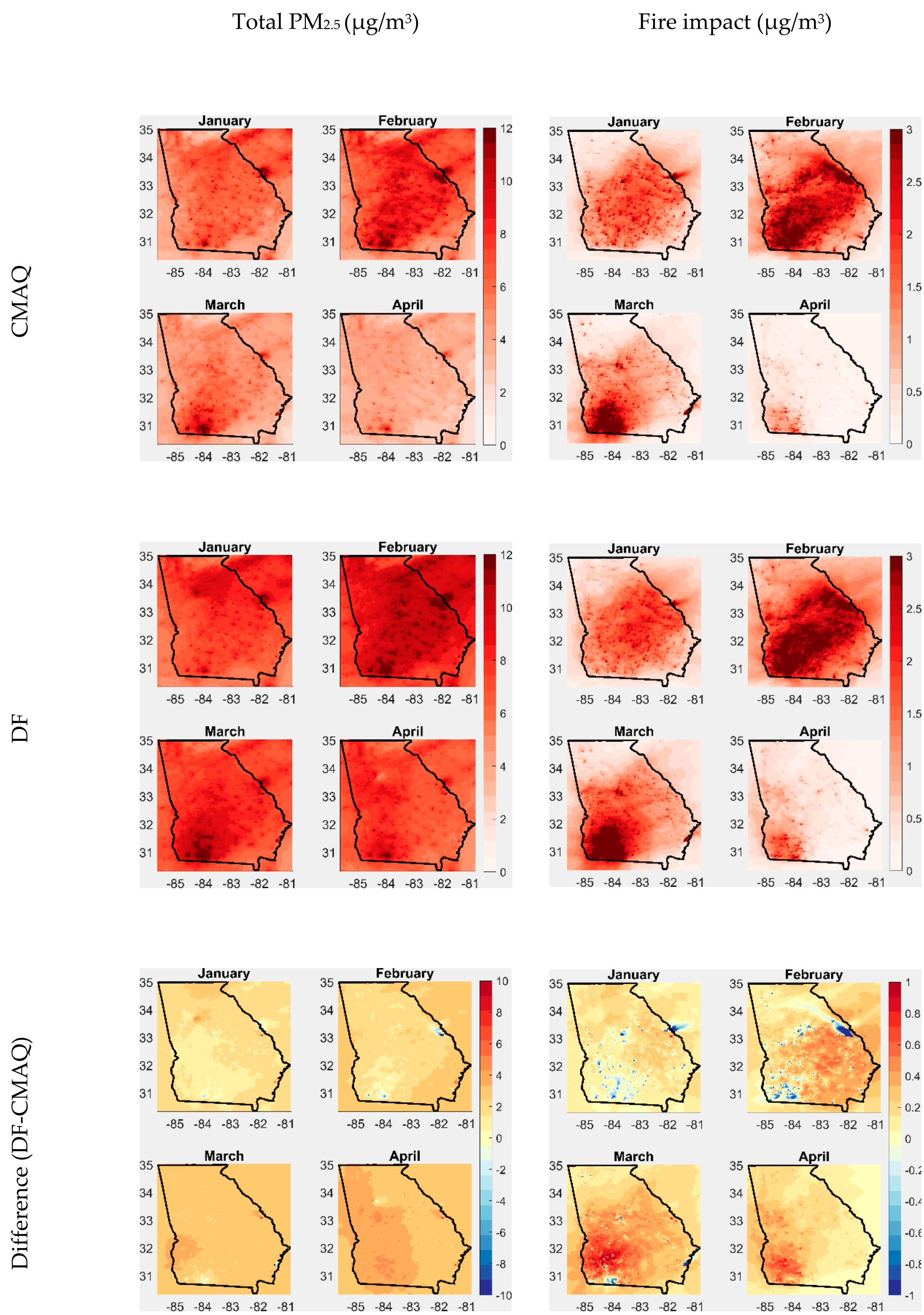


**Figure S3.** January–April monthly averages of total $PM_{2.5}$ and fire impact (2017): CMAQ-simulated, data fused and their difference.

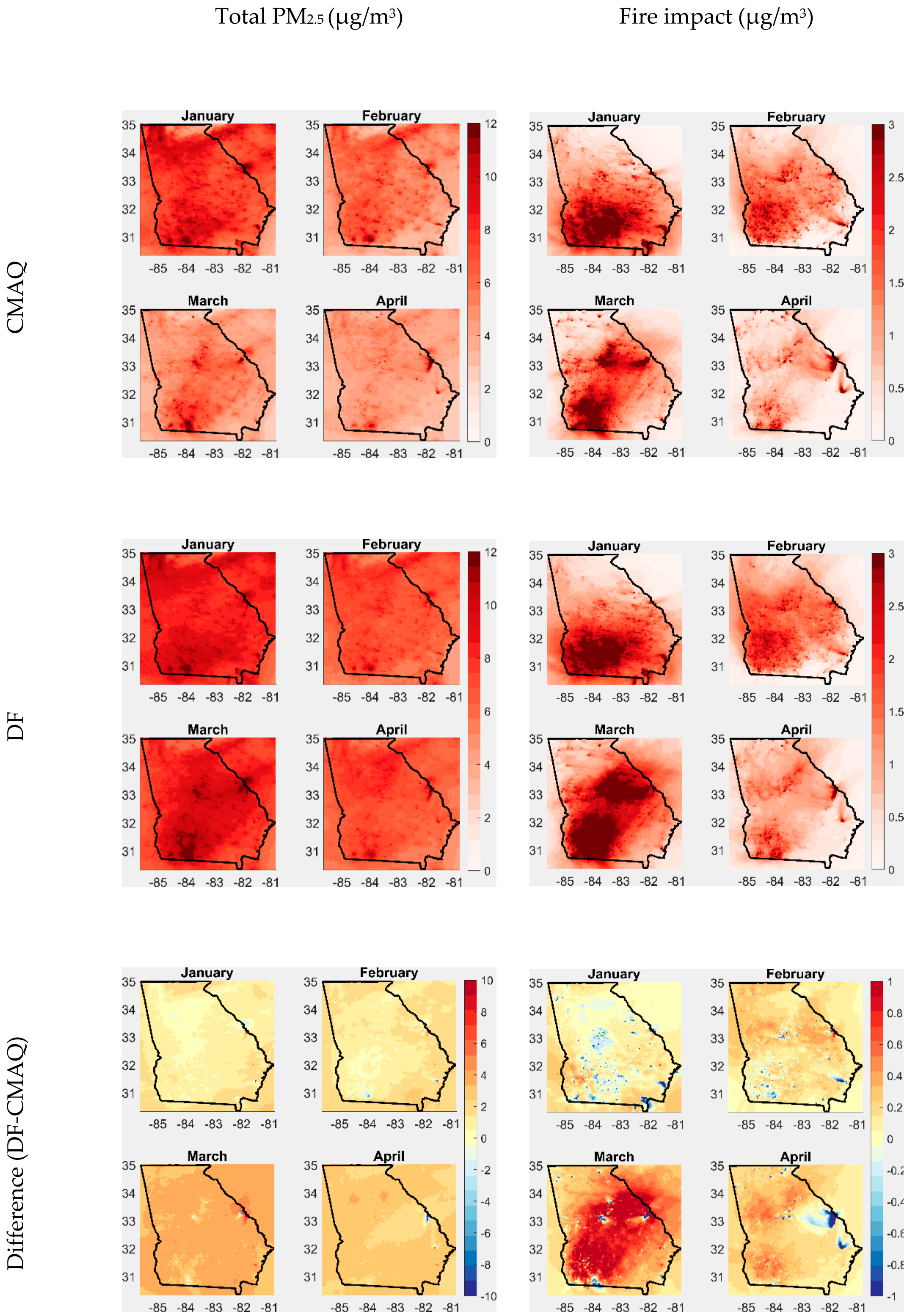


**Figure S4.** January–April monthly averages of total $PM_{2.5}$ and fire impact (2018): CMAQ-simulated, data fused and their difference.

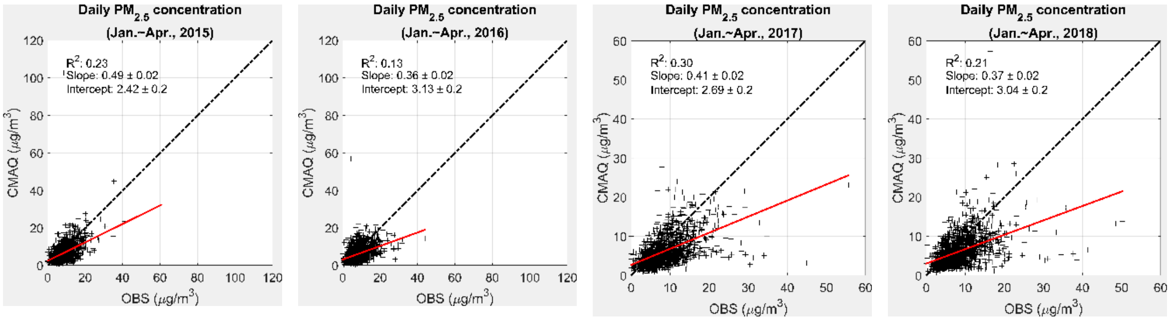


**Figure S5.** Comparison of daily total PM2.5 concentrations between observations (OBS) and CMAQ from 2015 to 2018, first four months.

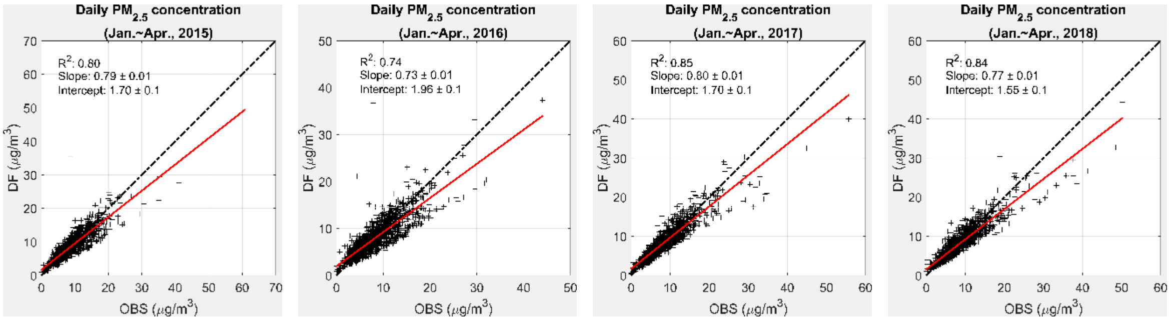


**Figure S6.** Comparison of daily total PM2.5 concentrations between observations (OBS) and DF from 2015 to 2018, first four months.

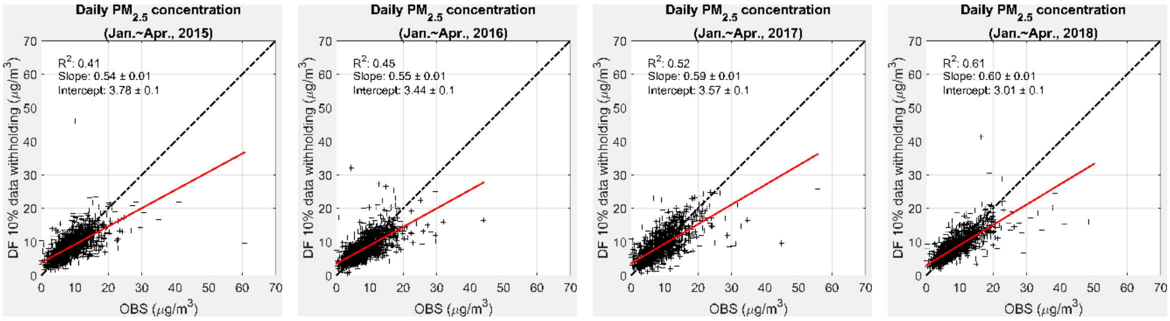


**Figure S7.** Comparison of daily total PM2.5 concentrations between observations (OBS) and 10% data withholding DF from 2015 to 2018, first four months.
